# Job design and behavioural outcome of employees in agricultural research training, Ibadan, Nigeria

**DOI:** 10.1016/j.dib.2018.06.073

**Published:** 2018-06-28

**Authors:** A.O. Osibanjo, A.J. Abiodun, O.P. Salau, A.A. Adeniji, H.O. Falola, I.I. Alimi

**Affiliations:** a Covenant University, Ota, Nigeria; b Federal University of Agriculture, Abeokuta, Nigeria

**Keywords:** Task identity, Autonomy, Feedback, Skill variety, Task significance, Behaviour, Design

## Abstract

This study focused on the relationship between job design and behavioural outcomes of employees in Agricultural Research Training, Ibadan, Oyo State, Nigeria. The study was quantitative and the items in the questionnaire were adapted from previous studies. A total of 227 respondents were surveyed and statistical regression models were used to examine the relationship between the independent variables (job design) and dependent variables (employee behavioural outcomes). The findings showed that 14.4% of the variance in job design dimensions can explain the variance in employee behavioural outcome. The model revealed that task identity, sense of autonomy and skill variety had more statistical significance in predicting employee behavioural outcome, recording the highest beta value than other variables such as task significance and feedback mechanisms. The model indicates that the strength of regression weights of paths has a strong direction.

**Specification Table**

**Table:** 

**Subject area**	Business, Management
**More Specific Subject Area:**	Strategic HRM
**Type of Data**	Primary data
**How Data was Acquired**	Through questionnaire
**Data format**	Raw, analyzed, Inferential statistical data
**Experimental Factors**	Population comprises employees in Agricultural Research Training. The researcher-made questionnaire contained data on job design and behavioural outcomes.
**Experimental features**	Influence of job design on behavioural outcome of employees
**Data Source Location**	Ibadan, Nigeria
**Data Accessibility**	Data is included in this article

**Value of data**

• The data can be used by government, private investors and other stakeholders to make decisions on how best to design and manage work activities and the impact of that on the work that people do.
• The data can be used to highlight the importance of changes that new technology is bringing to influence work design and the overall productivity of the institute.
• The data provides information on how different job designs dimensions can interact effectively to enhance positive behaviour and sustain greater commitment.
• Generally, data acquired from this study would be significant for improving institutional framework, facilitating goal achievement, and designing motivating work which would in turn lead to sustainability of the institute.

## 1 Data

The study is quantitative in nature and in an attempt to control for variability between companies (for example, how different companies simplify jobs, encourage skill variety, enrich the significance of the job, identify the meaningfulness of the work, responsibility for outcomes and knowledge of actual results), the scope of this study was limited to one research institute with multiple sections. The Job Characteristics Questionnaire (JCQ) developed by Hackman and Oldham was used to gather the core job characteristics data because it is the most validated and efficient means of accurately measuring job design.

Of the 300 JCQ surveys that were sent to the institute, 227 of these staff participated in the survey, for a 76% response rate. To achieve this response rate, several follow-up attempts were made to increase participation in the research. These included personal phone calls, as well as sending follow-up emails to the leaders asking for participation. After all data was collected, organized, and codified, it was analysed using SPSS, version 21. The independent variable for the study was job design. Data for the independent variable were collected via the JCQ survey instrument from voluntary participants within the company. Job design were measured using 15 questions. As recommended by the JCQ, the five measures and scales were used to calculate the score for each individual. These measures were identified as skill variety [SV], task identity [TI], task significance [TS], sense of autonomy [SA] and feedback mechanism [FM]. Cronbach׳s Alpha for job design was .869, well within the limits of acceptable reliability. Responses were collapsed by first averaging all items within each scale (three items each), then by averaging the resulting scores across all five scales to yield a single number representative of the level of job design for each respondent. In this study, the dependent variable was employee behavioural outcome as measured by employee engagement, job satisfaction and involvement in decision making. Thus, descriptive statistics for the indicators used in this analysis were not reported. The dependent variable data meet the assumptions for normality test.

During the data collection stage, demographic variables age, experience, and education were all coded, or scaled, so that the numbers shown do not reflect actual numbers. The scales used to code each of these variables is shown in Table 1.

**Table 1:** Demographic variable measurements.

Value	Education	Experience	Age	Marital Status	Gender
1	No formal education	<1 year	<20year	Single	Male
2	Primary education	1–5 years	21–30 years	Married	female
3	Secondary education	6–10 years	31–40 years	Divorced	
4	BSc./HND	>10 years	>40 years	Separated	
5	MSc./MEd.				
6	PhD.				

## 2 Data analysis

**Fig. 1:**
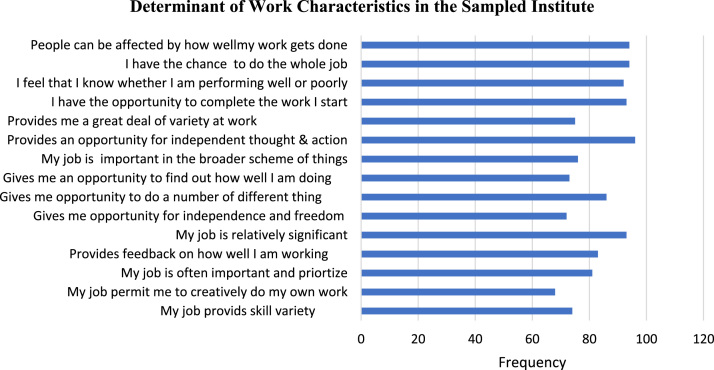
Determinant of work characteristics.

The study is quantitative in nature and data were retrieved from staff and management of sampled institute. The decision to elicit information from the employees and the management group was based on the fact that while employees were often in the best position to describe their job contents; it is also crucial to investigate these practice from the perceptions of the managers. This shows that the samples were diverse and it can be concluded that non-response bias will not significantly affect the generalizability of the study findings. The use of bar chart was also carried out to describe the work characteristics in the sampled institute as presented in Fig. 1.

**Fig. 2:**
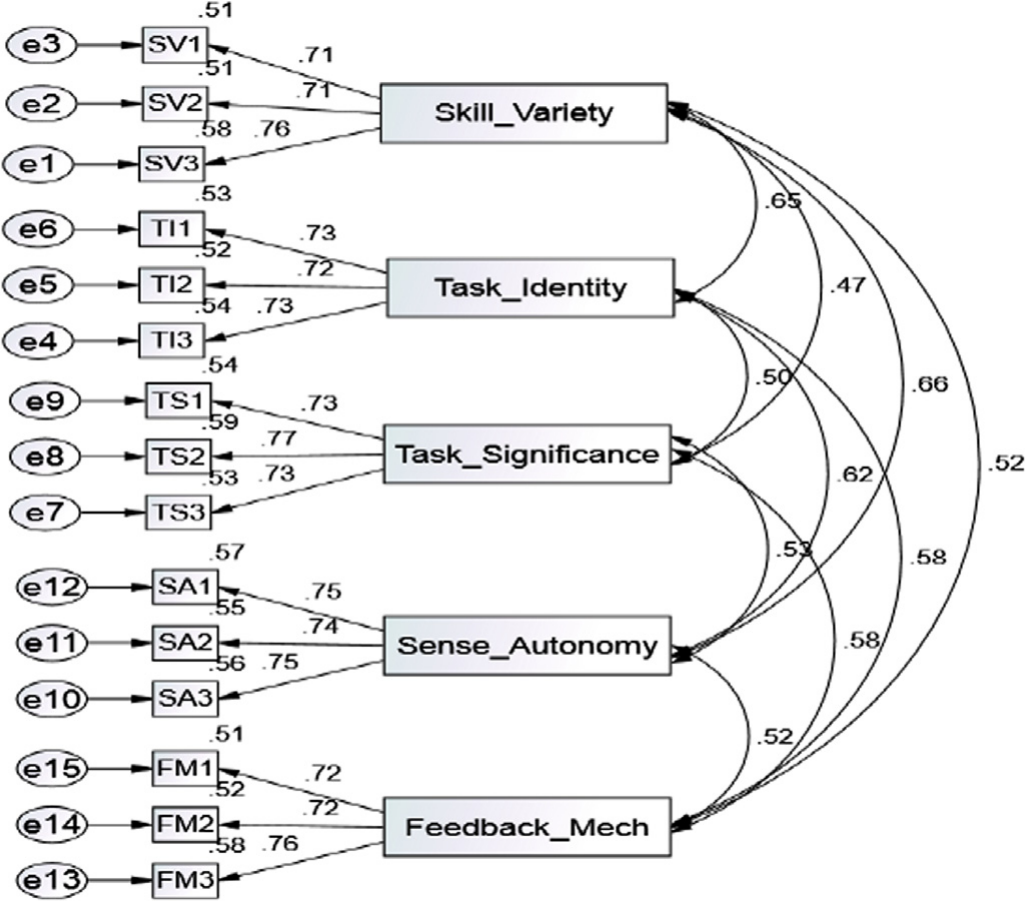
Confirmatory Factor Analysis.

The study adopted the approach recommended by [5] to evaluate: (1) measurement model and (2) structural model. To demonstrate the measurement model, we used Confirmatory Factor Analysis (CFA) and the three conditions for CFA loadings indicate that, first, all scale and measurement items are significant when it exceeds the minimum value criterion of 0.70. Second, each construct composite reliability exceeds 0.80. Third, each construct average variance extracted estimate (AVE) exceeds 0.50 as presented in Table 2 and Fig. 2 respectively.

**Table 2:** Reliability of the Instrument.

**Measurement**	**Loading**	**Indicator Reliability**	**Error Variance**	**Compose Reliability**	**Ave. Variance Estimated**
**Job Design**	**>0.7**		**<0.5**	**>0.8**	**>0.5**

a. **Skill Variety [SV]**					
SV1: Provide variety	0.823	0.6773	0.3227	0.8730	0.6962
SV2: Opportunity to do different things	0.835	0.6972	0.3028		
SV3: Provides variety at work	0.845	0.7140	0.2860		

b. **Task Identity [TI]**					
TI1: Opportunity to supervise Jobs/projects	0.828	0.6856	0.3144	0.8731	0.6966
TI2: Opportunity to complete work	0.864	0.7465	0.2535		
TI3: Opportunity to do whole job	0.811	0.6577	0.3423		

c. **Task Significance [TS]**					
TS1: Relatively significant in organization	0.876	0.7674	0.2326	0.8786	0.7074
TS2: Important in broader scheme	0.793	0.6288	0.3712		
TS3: People are affected by how well work gets done	0.852	0.7259	0.2741		

d. **Sense of Autonomy [SA]**					
SA1: Permit own work	0.805	0.6480	0.3520	0.8876	0.6639
SA2: Opportunity for independence and freedom	0.828	0.6856	0.3144		
SA3: Opportunity for self-thought and action	0.815	0.6642	0.3358		

e. **Feedback Mechanism** [**FM**]					
FM1: Provides feedback on work	0.805	0.6480	0.3520		
FM2: Opportunity to find out welfare	0.828	0.6856	0.3144		
FM3: Provides feeling for poor/good performance	0.815	0.6642	0.3358

Table 2 demonstrated convergent reliability, the researchers used CFA to assess composite reliability and the average variance extracted (AVE) of the specific constructs.

The results of CFA analysis suggest that the factor loadings for all major variables range between 0.704 and 0.761. The three conditions used to assess convergent validity as suggested and recommended [5], [8], [11] were met. After CFA analysis was conducted on the research model and the results indicate that the model fit the validity of the measurement, there is a need to re-examine the validity of constructs through discriminant validity test as recommended by [5], [12] For discriminant validity to be met, the square root of AVE for each construct should surpass the correlation of that construct and any other constructs. The discriminant validity was conducted using Pearson Correlation Matrix. As a threshold, the discriminant validity measurement should not be more than 0.90. Details of the results are available in Table 3, which exhibit that the coefficient correlation is highly correlated and are all significant.

**Table 3:** Discriminant validity.

**Correlations**							
**Items**		Skill_	Task_	Task_	Sense_	Feedback_	Emp_Beh_
		Variety	Identity	Sig.	Autonomy	Mech	Outcm
Skill_Variety	r	1	.653**	.467**	.661**	.515**	.284**
Task_Identity	r	.653**	1	.499**	.624**	.584**	.385**
Task_Significance	r	.467**	.499**	1	.534**	.581**	.245**
Sense_Autonomy	r	.661**	.624**	.534**	1	.523**	.288**
Feedback_Mech	r	.515**	.584**	.581**	.523**	1	.301**
Emp_Beh_Outcm	r	.284**	.385**	.245**	.288**	.301**	1

The diagonal values represent the square root of the average variance extracted (AVE) of the specific construct.

** . Correlation is significant at the 0.01 level (2-tailed).

**Fig. 3:**
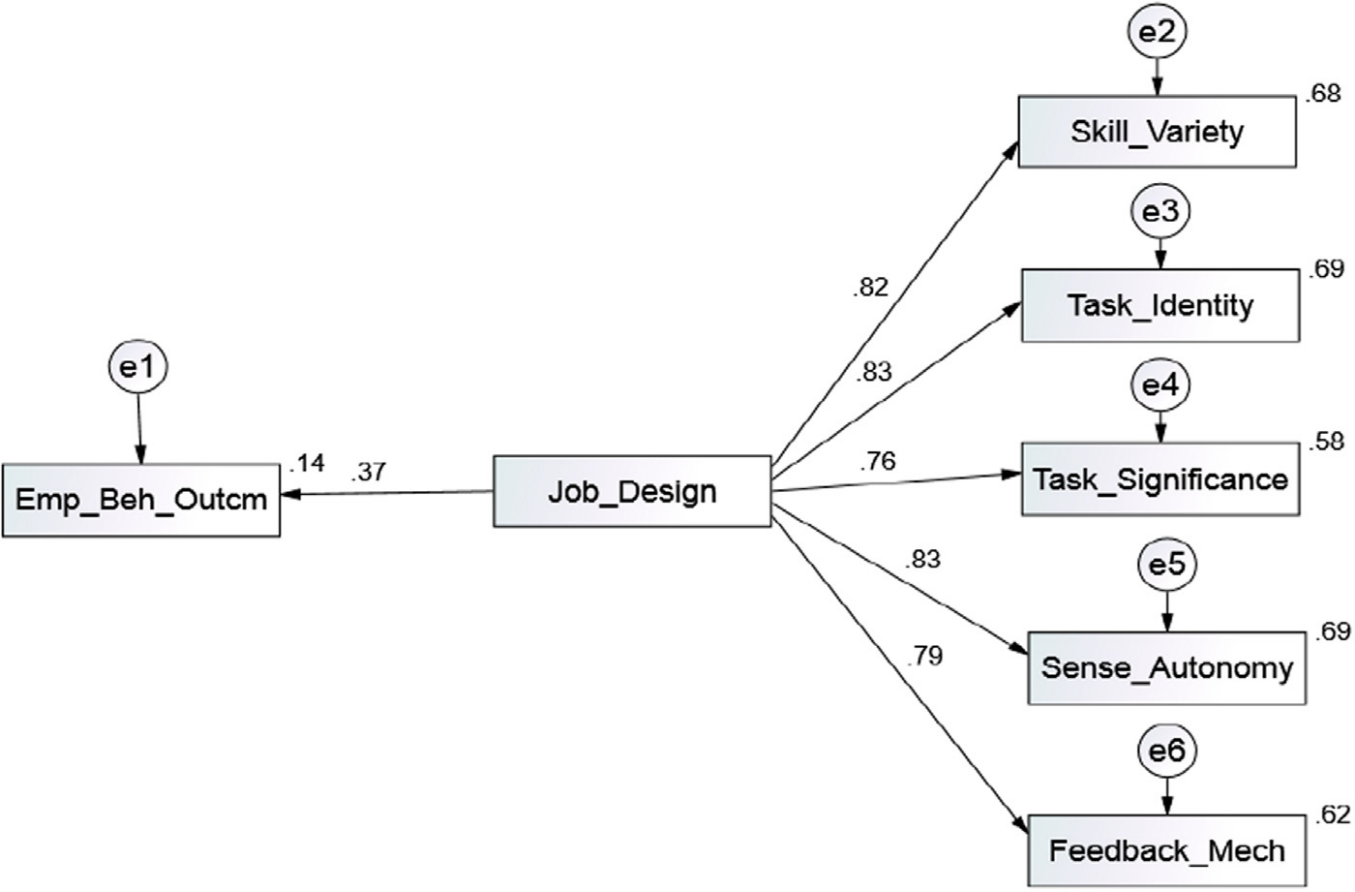
Job Design and Employee Behavioural Outcomes Model.

Based on the results of the test, it has proven that the data are good in terms of convergent validity, construct reliability, and discriminant validity. Moreso, a model fit was evaluated to show the relationship between observed and unobserved variables by examining several fit indices which include: chi-square (χ2), chi-square/degree of freedom (χ2/df), Goodness-of-Fit Index (GFI), Comparative Fit Index (CFI), Standardized Root Mean Residual (SRMR) and Root Mean Square Error of Approximation (RMSEA). Having run the test, the SEM was obtained, and results of fit indices was shown in Table 4 and Fig. 3.

**Table 4:** The Model Fit Summary Showing the Goodness of Fitness.

Goodness of fit	SEMs Value	Recommendation Values	Remarks
Chi­Square/Degree of Freedom (CMIN/DF)	2.524	≤3.00	Acceptable fit
Normed Fit Index (NFI)	0.973	≥.90	Good fit
Comparative Fit Index ( CFI)	0.942	≥.90	Very Good fit
Incremental Fit Index (IFI)	0.961	≥.90	Good fit
Root Mean Squared Error of Approximation (RMSEA)	.066	≤.08	Good fit
Goodness of Fit (GFI)	.935	≥.90	Good fit

Results in Table 4 dictate that the value of χ2/22=2.524, which is within the acceptable range between 1 and 3 [5], [12]. The value of RMSEA is 0.066, which is considered satisfactory (less than 0.08) as suggested by [1], [2], [3], [4], [11]. On top of that, the incremental fit, NFI, TLI, CFI, and GFI were above 0.90 as suggested by [5], [13]. Based on the results, it can be concluded that the overall fit indices are satisfactory. Meanwhile, results for standardised regression weights for each variable are stated in Table 5. It is seen that the strength of regression weights of paths has a strong direction.

**Table 5:** Standardised regression weights.

***Dependent***		***Independent***	**Estimate**	**S.E.**	**C.R.**	**P**	**Decision**
Emp_Beh_Outcm	<---	Job_Design	.372	.046	6.030	***	Significant
Skill_Variety	<---	Job_Design	.822	.049	21.675	***	Significant
Task_Identity	<---	Job_Design	.834	.046	22.691	***	Significant
Task_Significance	<---	Job_Design	.761	.053	17.644	***	Significant
Sense_Autonomy	<---	Job_Design	.829	.046	22.287	***	Significant
Feedback_Mech	<---	Job_Design	.790	.049	19.354	***	Significant

Before conducting the structural model, the data was tested for linearity, normality and multi-collinearity. All the basic assumptions were acceptable and prove that the data met the conditions of basic assumption in regression analysis. In this case, the R^2^=.144, which connotes that 14.4% of the variance in job design dimensions can explain the variance in employee behavioural outcome. This means that a unit increase in job design dimensions will lead to an increase in employee behavioural outcome. The model revealed that task identity, sense of autonomy and skill variety had more statistical significance in predicting employee behavioural outcome, recording the highest beta value than other variables such as task significance and feedback mechanisms. The model indicates that there are varying explanations for the dependent variables. Hence, it is seen that the strength of regression weights of paths has a strong direction.

## 3 Experimental design, materials and methods

Of 300 copies of questionnaire were distributed, only 227 responses were received resulting in a response rate of 76%. Data were gathered from directors, managers, assistant managers, scientists, field agents, and other categories of employees across the sampled institute with the aid of a researcher- made questionnaire based on the works of [6], [7], [9], [10], [13], [14], [15]. The demographic data presented information based on gender, age, education and experience as well as questions related to job design and employee behavioural outcome. The collected data were coded and analysed using SPSS version 22. Data was analysed through the measurement model and structural model. Importantly, the participants were selected based on the following inclusion criteria:

**Inclusion criteria:**

• Participants had to be staff of the sampled Institute
• Participants must have signed the consent form provided
• Participants must have worked with the institute for a minimum period of 3 years

However, the researchers ensured that respondents were well informed about the background and the purpose of this research and they were kept abreast with the participation process. Respondents were offered the opportunity to stay anonymous and their responses were treated confidentially.
