# Supplementary material for: Job design and behavioural outcome of employees in agricultural research training, Ibadan, Nigeria
Source: Data Brief. 2018 Jun 28;19:1880–7. doi: 10.1016/j.dib.2018.06.073 (PMC6141367; doi:10.1016/j.dib.2018.06.073)
Supplement: Supplementary file 1 — Supplementary material [file mmc1.doc]

**Falola Hezekiah Olubusayo (Ph.D)**

Department of Business Management

College of Business and Social Sciences,

Covenant University, Ota, Ogun State, Nigeria

[hezekiah.falola@covenantuniversity.edu.ng](mailto:hezekiah.falola@covenantuniversity.edu.ng)

+234 703 5518 559

**May 30, 2018**

The Editor,

Data in Brief

Dear Sir,

**DECLARATION OF CONFLICT OF INTEREST**

I, Dr. Falola H.O and my colleagues write to declare that there is no conflict of interest traceable to our data paper “Job design and behavioural outcome of employees in agricultural research training, Ibadan, Nigeria”

Thank you.

Yours faithfully,


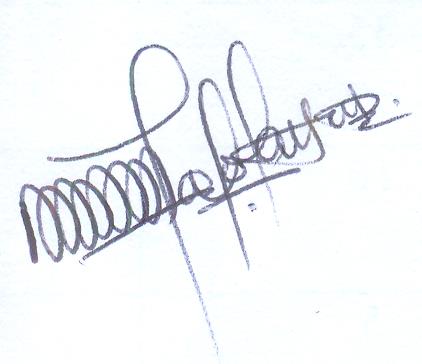


FALOLA H.O (PhD)

**Corresponding Author**
